# First simultaneous identification of *Moraxella bovoculi* and *Moraxella bovis* in one-humped camel (*Camelus dromedaries*) in the Sahara Desert

**DOI:** 10.1007/s11250-025-04836-3

**Published:** 2026-01-13

**Authors:** María de los Ángeles Ramo, Saleh Mohamed Lamin, Lochaa Mustafa, Embarek Mohamed Salem, Mohamed Chacha, Mariam Salma Muftah, Maite Rodríguez, Jaime Aranda, Alba Solsona, Luis Alcaraz-Rico, José-Alfonso Abecia

**Affiliations:** 1 Departamento de Patología Animal, Facultad de Veterinaria, Zaragoza, Spain; 2 Ministry of Public Health, Sahrawi Refugee Camps, Tindouf, Algeria; 3 Instituto Universitario de Investigación en Ciencias Ambientales de Aragón (IUCA), Universidad de Zaragoza, Zaragoza, Spain

**Keywords:** One-humped camel, *Moraxella*, Sahara desert, Keratoconjunctivitis

## Abstract

This study describes the first simultaneous identification of *Moraxella bovoculi* and *Moraxella bovis* in dromedary camels from the Sahrawi refugee camps in Tindouf, Algeria. Four lactating camels showing ocular lesions including conjunctival hyperemia, lacrimation, keratoconjunctivitis, and inflammation of the third eyelid were examined. Ocular swabs were collected and analyzed using real-time PCR for major bacterial and viral ocular pathogens. Only *M. bovoculi* (Cq 27) and *M. bovis* (Cq 36) were detected, while all other agents tested negative. Clinical recovery was achieved in all animals after topical treatment with oxytetracycline. The findings suggest a possible polymicrobial etiology, as has been described in cattle, and highlight the susceptibility of camels to pathogens traditionally associated with infectious bovine keratoconjunctivitis. The absence of other pathogens reinforces the role of *Moraxella spp*. as primary etiological agents in these cases. This report provides new insights into camel ocular health and emphasizes the importance of early diagnosis and treatment under field conditions. Moreover, it underscores the need to strengthen disease surveillance and preventive strategies in humanitarian contexts, where camel health is closely linked to the food security and livelihoods of displaced populations.

## Introduction

The study of animal pathologies in the Sahrawi refugee camps is of critical importance due to the vital role livestock plays in the livelihoods and food security of these communities. In such camps, where resources are scarce, and access to veterinary services is limited, animals provide essential contributions in the form of milk, meat, and trade, forming a cornerstone of both nutrition and economic stability (White 2018). However, the harsh desert environment, combined with constrained access to healthcare for animals, exposes livestock to a range of diseases, many of which can spread rapidly due to poor infrastructure and limited preventive measures. Additionally, zoonotic diseases—those transmissible between animals and humans—pose a significant public health risk in these densely populated camps, further highlighting the necessity of understanding and mitigating animal diseases (Braam et al. 2021).

Studying animal pathologies in this context offers the opportunity to develop targeted interventions to improve animal health and productivity, thereby enhancing food security, livelihoods, and overall community resilience (Rich and Perry 2011). Moreover, it contributes to global efforts to monitor and control emerging diseases in marginalized and vulnerable populations. Such research can also inform the design of sustainable veterinary programs, adapted to the unique challenges of refugee camp environments.

Certain ocular diseases in camels can be zoonotic, such as those caused by *Chlamydia spp.*, *Brucella spp.*, or *Coxiella burnetii*, may have zoonotic potential, meaning they can be transmitted to humans (Al-Salihi 2018). This is particularly concerning in densely populated areas like refugee camps where camels and humans interact closely. Monitoring and treating these diseases helps to protect human health as well.

Previous reports have documented infections by *Moraxella spp*. in camelids, highlighting their potential role in keratoconjunctivitis in this host. Outbreaks of ocular disease associated with a specific biovar of *Moraxella canis* were described in dromedary camels in the Canary Islands, demonstrating that multiple *Moraxella* species may act as primary ocular pathogens in camels (Tejedor-Junco et al. 2010). More recently, *Moraxella bovoculi* has been identified in cases of infectious keratoconjunctivitis in one-humped camels, and antimicrobial susceptibility patterns have been reported, underscoring its emerging relevance in camel ocular health (Özavcı and Seferoğlu 2023). These studies indicate that camelids are susceptible to several *Moraxella spp*., and they provide important context for interpreting the detection of *M. bovoculi* and *M. bovis* in the present work.

In addition to *Moraxella spp*., several other bacterial and viral agents may contribute to ocular disease in camelids, and their diagnosis typically relies on molecular methods. Mycoplasma species, particularly *Mycoplasma conjunctivae* and *Mycoplasma bovis*, are recognized causes of keratoconjunctivitis in ruminants, and real-time PCR has become the preferred diagnostic tool due to its superior sensitivity compared with culture, which is often slow and difficult because of the fastidious nature of these organisms. *Chlamydiaceae spp*., another group of pathogens occasionally implicated in ocular infections in camelids and other livestock, are commonly detected using PCR assays targeting conserved regions of the chlamydial genome. Viral causes, such as bovine herpesvirus type 1 (BoHV-1), can also be identified through PCR, which allows sensitive detection even in mild or early infections. The availability of multiplex or panel-based qPCR platforms enables simultaneous screening of these pathogens, providing a comprehensive approach to the differential diagnosis of keratoconjunctivitis in field settings.

The objective of this humanitarian cooperation conducted in the Sahrawi refugee camps, focused on camels with ocular lesions, is to identify, analyze, and address the underlying causes of these eye conditions to improve the health and well-being of the camels. Camels are a critical resource for the Sahrawi population, providing transportation, food (milk and meat), and economic sustenance in the harsh desert environment.

## Materials and methods

This work is part of a humanitarian cooperative project funded by the University of Zaragoza and the Government of Aragón (Spain), with the objective of diagnose, control and prevent transmissible zoonoses in the Sahrawi refugee camps in Tindouf (Algeria), following the guidelines of the so-called One Health strategy.

### Animals

**Fig. 1:**
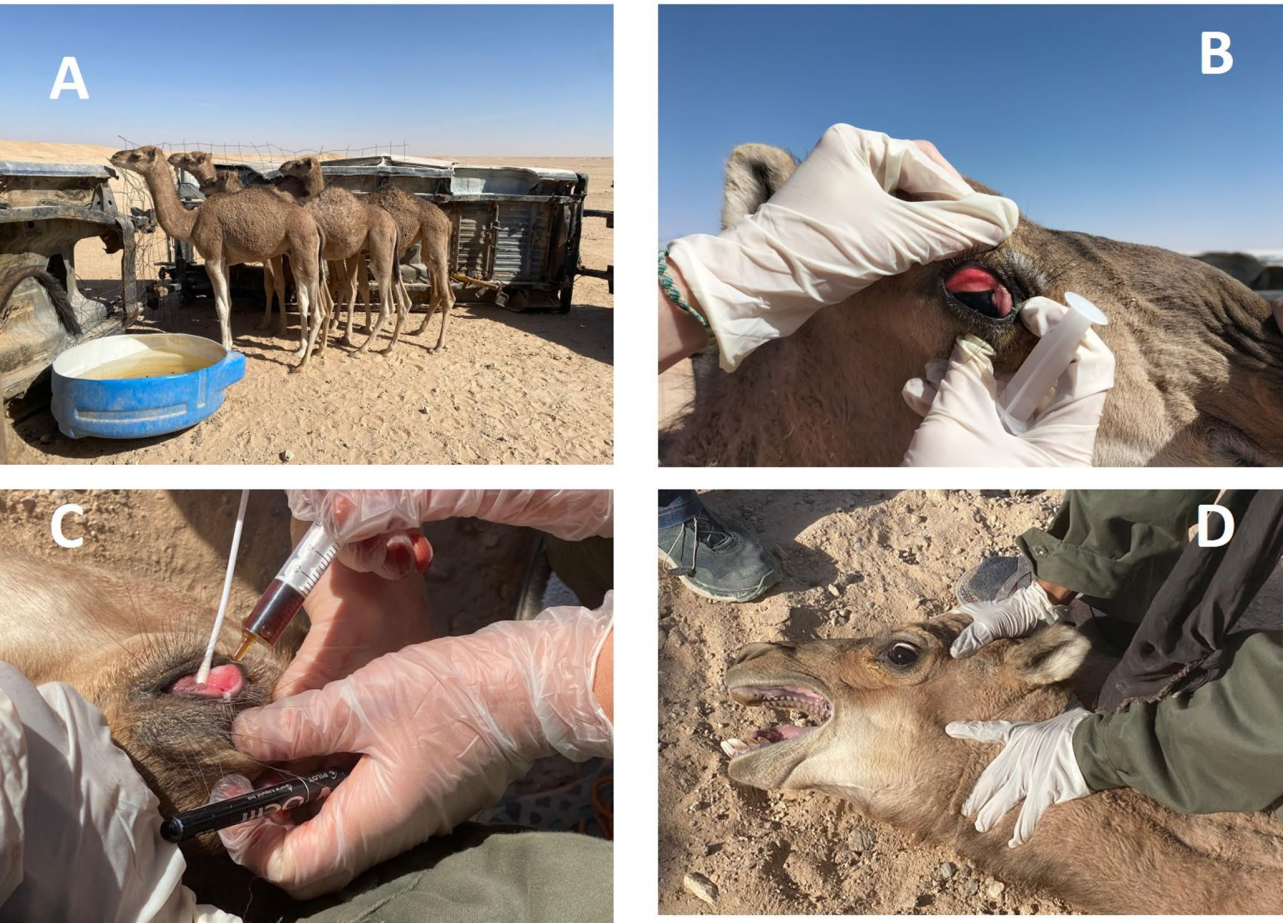
Clinical signs and evolution of ocular disease in dromedary camels from the Sahrawi refugee camps. (**A**) Affected lactating camel from the herd. (**B**) Clinical presentation of ocular lesions showing conjunctival hyperemia, lacrimation, and inflammation of the third eyelid. (**C**) Collection of ocular swab samples for microbiological analysis and treatment with oxytetracycline. (**D**) Resolution of lesions after treatment with oxytetracycline

This study was carried out in December, at the Smara wiolayat of the Sahrawi Refugee Camps (27°29′N, 7°49′W) located in Tindouf province in southwest Algeria. Four lactating dromedary camels (Fig. 1A) were selected from a herd, with a history of blindness, separation from their mothers, disorientation and weight loss. The camels were physically restrained by a skilled operator with the least possible stress to the animal. General clinical and ocular examination were conducted on each camel. The four animals presented clinical signal of conjunctival hyperemia, keratoconjunctivitis, lacrimation signals and inflammation of the third eyelid (Fig. 1B). One of the animals presented these lesions in one eye, only.

### Sample collection

One swab sample was collected from the affected eyes (Fig. 1C), and were submitted to Exopol (San Mateo, Zaragoza, Spain) for a bacteriological examination, as a pool. Samples were pooled because field conditions, limited laboratory capacity in the refugee camps, and the humanitarian nature of the mission required minimizing the number of analyses. Additionally, the study was exploratory and diagnostic rather than epidemiological, and individual identification of the camels was not feasible under the field circumstances, as animals could not be reliably tagged or tracked during the visit.

### Real-time PCR identification of ocular pathogens

Molecular detection of ocular pathogens was carried out using commercial EXOone qPCR kits (Exopol, Zaragoza, Spain) specific for *Moraxella bovis* (EXOone *Moraxella bovis*, ref. MRXB), *Moraxella bovoculi* (EXOone *Moraxella bovoculi*, ref. MBUC), *Mycoplasma bovis*,* Mycoplasma conjunctivae*,* Chlamydiaceae spp.*, and bovine herpesvirus type 1 (BoHV-1), following the manufacturer’s instructions. Each reaction included the primers and probe supplied within the kit, the corresponding qPCR master mix, and 5 µL of template DNA in a final reaction volume of 20 µL. The kits incorporate an endogenous internal control, as well as a positive control and a no-template negative control, which were included in every run to validate amplification efficiency and to rule out contamination. Primer and probe sequences are proprietary and were not disclosed by the manufacturer. Amplification was performed in a QuantStudio 5 Real-time PCR machine (Applied Biosystems, Marsiling, Singapore) using the cycling conditions recommended by Exopol. Samples with a quantification cycle (Cq) ≤ 38 were considered positive.

### Treatment and control of the animals

The animals were treated with 4 ml of oxytetracycline dihydrate 20% (Argelian Animal Health Products) by the ocular route. After five days, animals were restrained and their lesions checked (Fig. 1D).

## Results and discussion

The results of the Real-Time PCR are shown in Table 1. Out of the eight pathogens tested by qPCR, only *Moraxella bovis* and *Moraxella bovoculi* were detected in the samples. *Moraxella bovis* showed a high quantification cycle (Cq) value of 36, indicating a lower bacterial load, whereas *Moraxella bovoculi* was detected with a Cq value of 27, suggestive of a comparatively higher bacterial presence. All other agents—including *Moraxella ovis*,* Mycoplasmopsis bovis*,* Mesomycoplasma bovoculi*,* Mesomycoplasma conjunctivae*,* Chlamydiaceae spp.*,* and bovine herpesvirus type 1* (IBR)—tested negative, with no amplification observed.

**Table 1:** Results of real-time PCR analysis of ocular swabs from four dromedary camels with ocular lesions in the Sahrawi refugee camps. *Moraxella Bovis* and *Moraxella bovoculi* were detected, whereas other tested pathogens were negative

Bacteria	Result	Cq
*Moraxella bovis*	Positive	Cq 36
*Moraxella bovoculi*	Positive	Cq 27
*Moraxella ovis*	Negative	-
*Mycoplasmopsis bovis.*	Negative	-
*Mesomycoplasma bovoculi*	Negative	-
*Mesomycoplasma conjunctivae*	Negative	-
*Chlamydiaceae (sp.)*	Negative	-
*Herpesvirus bovino tipo 1 (IBR)*	Negative	-

At presentation, all four lactating camels showed conjunctival hyperemia and moderate to marked lacrimation (Table 2). Three animals exhibited varying degrees of corneal opacity, while one animal showed unilateral involvement with mild corneal edema. No precise measurements of corneal ulcer size were possible under field conditions; however, based on visual inspection, none of the animals presented deep or perforating ulcers, and no stromal melting was observed. After five days of topical oxytetracycline administration, all animals showed marked improvement, with resolution of hyperemia and lacrimation, and progressive clearing of corneal opacity. The animals presented a successful clinical recovery after the treatment, with no signs of blindness (Fig. 1D).

**Table 2:** Clinical findings of ocular lesions in four dromedary camels at presentation and after treatment

Animal	Eye(s) affected	Initial clinical signs	Corneal opacity	Corneal ulceration	Lesions after treatment (Day 5)
1	Bilateral	Hyperemia, lacrimation	Moderate	Not measurable; superficial appearance	Lesions resolved; cornea clearing
2	Bilateral	Hyperemia, edema	Mild–moderate	Not measurable; no deep ulcer	Complete resolution
3	Unilateral	Hyperemia, lacrimation	Mild	No obvious ulcer	Complete resolution
4	Bilateral	Hyperemia, marked lacrimation	Moderate	Not measurable	Significant improvement; no blindness

This study reports the first simultaneous detection of *Moraxella bovoculi* and *Moraxella bovis* in dromedary camels in the Sahrawi refugee camps of Tindouf, Algeria. The ocular lesions observed—conjunctival hyperemia, lacrimation, keratoconjunctivitis, and third eyelid inflammation—are compatible with infectious keratoconjunctivitis (IBK), a disease extensively described in cattle but poorly characterized in camels. The identification of both *M. bovis*, a well-established pathogen of IBK, and *M. bovoculi*, increasingly reported in ocular infections of ruminants, suggests that camels may also be susceptible to these agents (Angelos 2015).

The detection of both species in the same pooled sample points towards a possible polymicrobial etiology. In cattle, co-infections of *M. bovis* and *M. bovoculi* have been associated with more severe or persistent clinical outcomes (Szacawa et al. 2015). Whether a similar synergism occurs in camels remains unknown, but this finding highlights the need to further investigate the ocular microbiome of camelids, especially in environments characterized by nutritional stress and limited access to veterinary care.

The absence of other pathogens commonly associated with ocular disease in ruminants, such as *Mycoplasmopsis bovis*,* Mesomycoplasma conjunctivae*,* Chlamydiaceae* spp. or bovine herpesvirus type 1, reinforces the role of *Moraxella* spp. as the primary etiological agents in these cases. Environmental and management factors, including close contact among animals, shared water sources, and the presence of mechanical vectors such as flies, may contribute to the transmission and maintenance of these bacteria in camel herds (Higgins 1986).

Although corneal ulcer size is an important indicator of chronicity and disease progression in infectious keratoconjunctivitis (Angelos 2015), precise measurements could not be obtained due to field limitations and the need to minimize animal handling during the humanitarian visit. As a result, our description of ocular lesions is based on clinical impressions and photographic evidence rather than standardized ophthalmic metrics. This limitation restricts our ability to precisely characterize the severity of lesions at presentation. Nevertheless, the rapid clinical improvement observed after topical oxytetracycline suggests that the animals were likely treated during the early stages of the disease, when therapeutic response is most favorable.

It is also important to note that the use of pooled samples represents a methodological limitation. Because individual ocular swabs could not be processed separately, it is not possible to determine whether both pathogens were present in all animals. Therefore, while the co-detection of *M. bovoculi* and *M. bovis* suggests potential polymicrobial involvement, mixed etiology cannot be confirmed. This limitation reflects the field conditions of the humanitarian mission, where individual sampling and repeated handling of animals were not feasible.

The favorable clinical response to topical oxytetracycline confirms the effectiveness of antimicrobial therapy when applied early, even under field conditions. Nevertheless, limitations exist in free-ranging camels, where re-treatment and close monitoring may be difficult. For this reason, preventive measures should also be emphasized, such as improving environmental hygiene, controlling vectors, and raising awareness among herders.

From a broader perspective, this study underlines the importance of applying a One Health approach in humanitarian contexts. Camel health directly impacts the nutrition, mobility, and livelihoods of displaced Sahrawi communities, and ocular diseases reduce milk production, transport capacity, and overall animal welfare. Integrating molecular diagnostic tools such as qPCR into humanitarian veterinary programs, although logistically challenging, can significantly enhance disease surveillance and local capacity-building (Destoumieux-Garzón et al. 2018).

## Conclusion

In conclusion, the simultaneous presence of *M. bovoculi* and *M. bovis* in camels suggests that both bacteria may play a role in ocular pathology in this species. Future work should expand surveillance to larger camel populations, explore possible cross-species transmission with cattle and small ruminants, and evaluate preventive strategies adapted to the socio-ecological conditions of long-term refugee settlements.

## Data Availability

Data are available from the corresponding author upon reasonable request.
